# Beyond Weight Loss: A Comprehensive Review of Integrated Pharmacotherapy and Metabolic Bariatric Surgery in Endometrial Cancer Care

**DOI:** 10.3390/medicina62081571

**Published:** 2026-08-17

**Authors:** Chiara Innocenzi, Marta Goglia, Elisa Reitano, Matteo Pavone, Giorgio Fiorenza, Michela Orsi, Denis Querleu, Mariano Eduardo Giménez, Gianfranco Silecchia, Nicolò Bizzarri, Anna Fagotti, Francesco Fanfani, Jacques Marescaux, Antonello Forgione

**Affiliations:** 1 UOC Ginecologia Oncologica, Dipartimento di Scienze Della Salute Della Donna e Del Bambino, Fondazione Policlinico Universitario A. Gemelli, IRCCS, 00168 Rome, Italy; 2 Università Cattolica del Sacro Cuore, 00168 Rome, Italy; 3 Research Institute Against Digestive Cancer (IRCAD), 67091 Strasbourg, France; 4 Department of Medical and Surgical Sciences and Translational Medicine, Faculty of Medicine and Psychology, Sapienza University of Rome, 00185 Rome, Italy; 5 Department of General Surgery, Nouvel Hôpital Civil (NHC), University of Strasbourg, 67000 Strasbourg, France; 6 Institute of Image-Guided Surgery, IHU Strasbourg, 67000 Strasbourg, France; 7 ICube, UMR 7357, CNRS, INSERM U1328 RODIN, University of Strasbourg, 67091 Strasbourg, France; 8 Department of Surgical Sciences, University of Rome Tor Vergata, 00133 Rome, Italy; 9 DAICIM Foundation, Teaching, Assistance and Research in Minimally Invasive Surgery, Department of General and Minimally Invasive Surgery, University of Buenos Aires, Buenos Aires 1014, Argentina

**Keywords:** bariatric surgery, bariatric endoscopy, endometrial cancer, metabolic syndrome, obesity, minimally invasive surgery

## Abstract

*Backgroundand Objectives*: Endometrial cancer ranks among the most prevalent gynecologic malignancies in high-income countries, with increasing incidence and mortality driven by the global obesity epidemic. Evidence suggests that women with early-stage disease face a greater risk of death from obesity-related comorbidities than from cancer recurrence and that weight reduction is associated with improvements in quality of life and in the long-term burden of obesity-related morbidity and mortality. Minimally invasive (MIS) metabolic/bariatric surgery is the most effective treatment for achieving significant, sustained weight loss and comorbidities resolution/improvement. Advances in MIS (laparoscopic, robotic, and endoscopic) techniques have reduced perioperative morbidity and mortality. Novel pharmacotherapies, including glucagon-like peptide-1 receptor agonists (GLP-1RAs), have recently received regulatory approval for weight management and ongoing studies are investigating their therapeutic impact on endometrial carcinogenesis and tumor biology. This review synthesizes and critically appraises evidence on metabolic interventions, including innovative bariatric techniques and pharmacologic agents, with the aim of informing their integration into multidisciplinary, risk-adapted management strategies for patients with severe obesity and endometrial cancer. *Materials and Methods*: A comprehensive review was conducted in accordance with SANRA (Scale for the Assessment of Narrative Review Articles) criteria. *Results*: Emerging evidence indicates that MIS metabolic/bariatric surgery combined with hysterectomy is feasible and may be integrated into the multidisciplinary management of selected patients with severe obesity and endometrial hyperplasia or carcinoma. GLP-1RAs may modulate both the obesogenic systemic milieu and selected tumor-associated pathways, although definitive clinical evidence of direct antitumor effect and oncologic benefit in endometrial cancer has not been established. *Conclusions*: Obesity is a modifiable risk factor for endometrial cancer. Metabolic/bariatric interventions represent a promising component of the comprehensive, patient-centered management of this disease. Prospective studies are essential to clarify the association between these interventions and longitudinal oncologic outcomes, the feasibility and potential benefits of combined approaches, and their differential impact according to the molecular classification of endometrial cancers. Although weight reduction is associated with a lower risk of endometrial cancer, a direct improvement in cancer-specific survival has not yet been demonstrated; weight-loss management therefore represents a promising component of care in the severely obese population, and its incorporation into formal guidelines requires confirmation in prospective studies.

## 1. Introduction

Obesity currently affects 800 million people worldwide [1] and the association between excess body weight and increased cardiovascular and all-cause mortality is extensively documented, largely because of its link to various chronic diseases and cancer types [2]. Several factors, including inflammation, immunological reactions, insulin resistance, changes in the local microenvironment and hormone-related pathways, have been postulated to contribute to the detrimental effects of obesity on cancer development [3].

However, after diagnosis, the prognostic impact of a high body mass index (BMI) on oncological patients is unclear or even described as protective in some reports. Studies on head and neck, esophageal, breast, lung cancer and renal cell carcinoma documented an ‘obesity paradox’, whereby obese patients have superior survival outcomes [4]. Nevertheless, the biological and clinical interplay between excess body weight, cancer incidence and mortality remains poorly understood, as the available evidence is heterogeneous, sometimes contradictory or limited by methodological constraints [5].

Endometrial cancer (EC) represents a major global health concern, as it is the most frequently diagnosed gynecologic malignancy worldwide, particularly in high-income countries [6]. Metabolic syndrome and related conditions, obesity, diabetes, and ovarian dysmetabolic syndrome, are risk factors for its development [6]. In contrast, when considered as a prognostic factor, obesity is associated with a 44% reduction in 5-year overall survival among women diagnosed with EC, directly affecting survival outcomes independently of disease recurrence [6]. Beyond its medical and social implications, obesity also constitutes a major public health challenge due to its economic burden. Focusing on patients with EC, in whom surgery represents the mainstay of treatment [7], obesity further amplifies healthcare costs by increasing surgical complexity, length of hospital stay (LOS), and the risk of treatment-related morbidity [8].

Obesity should be considered not only a risk factor, but also a therapeutic target that can be incorporated into oncologic decision-making through risk-adapted metabolic interventions. Several evidence-based strategies proved efficacy in reducing obesity and its associated EC risk, including structured lifestyle intervention programs, metabolic bariatric surgery (MBS), and recent advances in pharmacotherapy, such as glucagon-like peptide-1 (GLP-1) receptor agonists (GLP-1RAs) [8]. Metabolic interventions may act at three distinct, although interconnected, levels in endometrial cancer care: risk reduction before cancer development, perioperative and survivorship optimization after diagnosis, and, more speculatively, modulation of tumor biology.

Tackling such a complex disease requires a multidisciplinary approach that spans medical and surgical subspecialties and is coordinated across the entire cancer care continuum. A comparable rationale has recently emerged for colorectal cancer, where synchronized management of severe obesity and oncologic treatment has been advocated, reinforcing the notion that obesity-driven malignancies need care pathways that concurrently address the underlying metabolic disease [9]. Accordingly, this comprehensive review aims to appraise the contemporary evidence on weight-loss interventions, including innovative bariatric surgical techniques and pharmacologic therapies, in patients affected by endometrial cancer and preneoplastic lesions and evaluate how these strategies can be rationally integrated into a personalized, multidisciplinary oncologic management framework.

## 2. Materials and Methods

This comprehensive review provides an updated overview of metabolic interventions in patients with EC and excess body weight, encompassing the spectrum of metabolic dysfunction from overweight to obesity. The methodology aligns with the SANRA (Scale for the Assessment of Narrative Review Articles) criteria [10]. A comprehensive literature search was performed in PubMed, Google Scholar, Scopus and Web of Science, covering publications from inception to June 2026, with an English-language restriction. The ClinicalTrials.gov registry was also searched to identify relevant ongoing and recently completed interventional and observational studies. The search combined terms including: (“endometrial cancer” OR “endometrial carcinoma” OR “endometrial hyperplasia”) AND (“obesity” OR “metabolic syndrome” OR “bariatric surgery” OR “metabolic surgery” OR “GLP-1 receptor agonist” OR “semaglutide” OR “liraglutide” OR “tirzepatide” OR “weight loss” OR “pharmacotherapy” OR “fertility-sparing”). Additional searches targeted specific subtopics: (“robotic bariatric surgery”), (“endoscopic sleeve gastroplasty”), (“combined hysterectomy bariatric surgery”), and (“GLP-1RA endometrial cancer”). Eligible sources included original research, randomized controlled trials (RCTs), systematic reviews, meta-analyses, narrative reviews and case series, case reports, economic models, and guideline documents. Evidence was prioritized in the following hierarchy: systematic reviews, meta-analyses and RCTs, followed by prospective cohort studies, retrospective studies, economic analyses, case series and reports, and finally preclinical and experimental data. No formal risk-of-bias assessment or meta-analysis was performed given the narrative design; conclusions were based on qualitative assessment.

## 3. Metabolic Disease and Endometrial Cancer: Epidemiology, Awareness and Healthcare Costs

BMI-based classification of obesity includes class I obesity (30–34.9 kg/m^2^), class II obesity (35–39.9 kg/m^2^), and class III or severe obesity (≥40 kg/m^2^) [9]. In 2016, approximately 59% of adults in the European Union (EU) were classified as having pre-obesity or obesity, resulting in an estimated total cost of €70 billion for that year, including healthcare expenses and lost productivity [11]. In the United States (U.S.), where obesity prevalence increased from 31% in 2000 to an estimated 42% in 2020, obesity was associated with more than $260 billion in annual healthcare spending in 2016 alone [2]. Endometrial cancer, in contrast, is the most common gynecologic cancer in high-income countries, and its incidence has increased globally by 132% over the past 30 years, reflecting a rise in the prevalence of risk factors, particularly obesity and an aging population [12]. Hence, it is estimated that approximately 50% of all new EC diagnoses are attributable to overweight and/or obesity [13].

**Figure 1:**
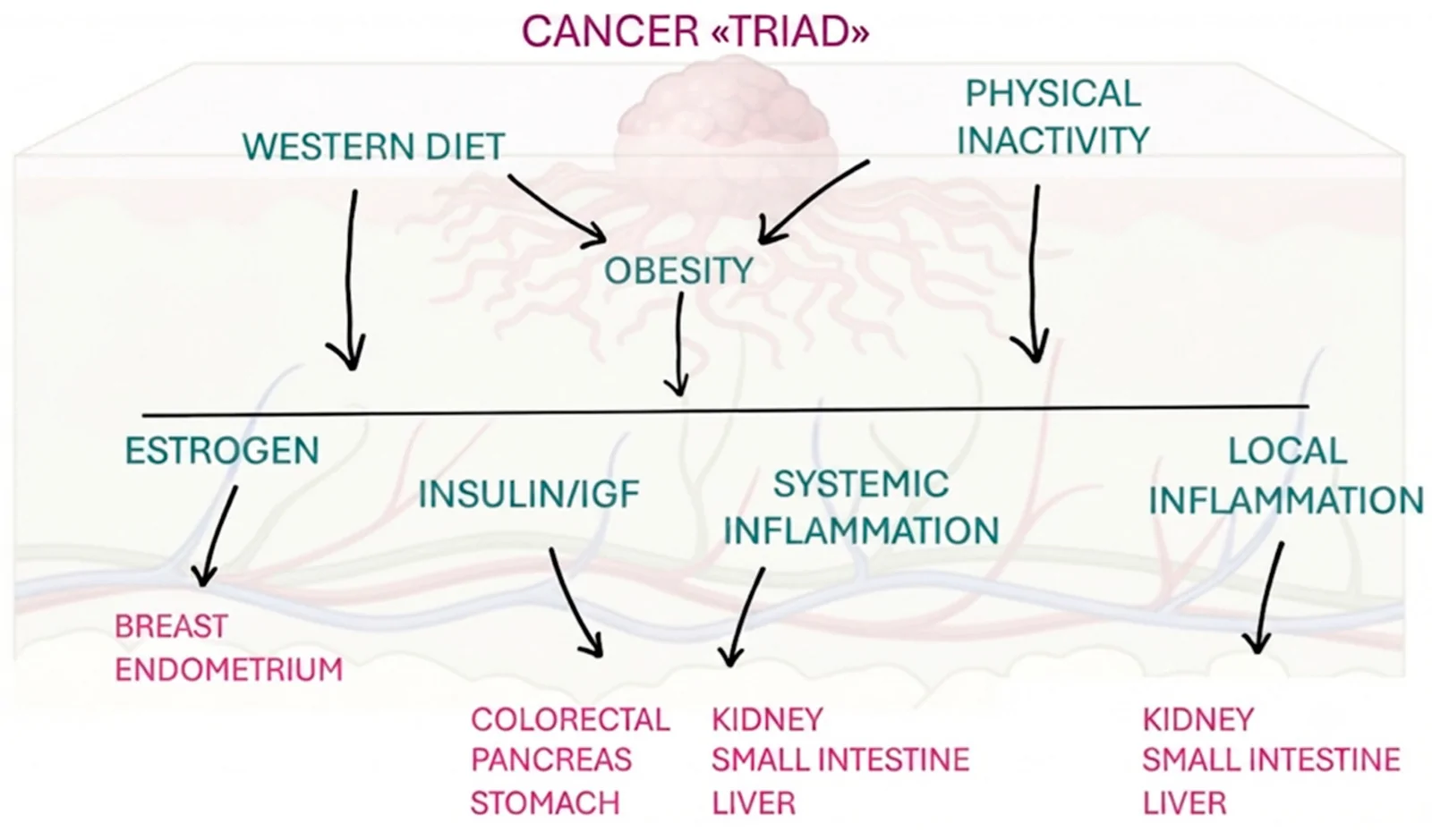
The “cancer triad.” Western diet, physical inactivity, and obesity act as interrelated risk factors that drive carcinogenesis across multiple anatomic sites through shared pathways: increased estrogen bioavailability (breast, endometrium), activation of the insulin/insulin-like growth factor (IGF) axis (colorectum, pancreas, stomach), and chronic systemic and local inflammation (kidney, small intestine, liver).

From a pathophysiological standpoint, obesity is an established driver of carcinogenesis that acts as the central node of a cancer triad in which a Western dietary pattern and physical inactivity converge with excess adiposity to promote malignant transformation across numerous anatomic sites [14] (Figure 1).

Among these malignancies, the endometrium is one of the most tightly linked to excess body weight. Excess adiposity promotes a chronic proinflammatory milieu characterized by elevated circulating levels of C-reactive protein (CRP), interleukin-6 (IL6), and tumor necrosis factor-α (TNFα), together with a relative depletion of protective immune cell populations within the endometrium. In parallel, obesity promotes a hyperestrogenic environment through the peripheral aromatization of adrenal androgens into estrogens by adipose tissue. Estrogen, in turn, stimulates endometrial proliferation; in postmenopausal women, the physiological deficiency of progesterone further exacerbates this effect by permitting sustained unopposed estrogen exposure [12]. Additionally, insulin resistance and compensatory hyperinsulinemia, hallmark features of obesity, type 2 diabetes mellitus (T2D), and ovarian dysmetabolic syndromes, enhance endometrial stimulation by increasing the bioavailability of both estrogen and insulin-like growth factor-1 (IGF-1). These hormonal and metabolic alterations converge on the activation of the pro-oncogenic PI3K-AKT-mTOR signaling pathway through both direct (estrogen-mediated) and indirect (IGF-1-mediated) mechanisms, thereby promoting the accumulation of unfavorable somatic genetic events and subsequent malignant transformation [3]. Risk-reducing interventions that modulate these pathogenic pathways, including weight reduction, increased physical activity, exogenous progestin therapy, and the use of anti-inflammatory and insulin-sensitizing agents, represent a rational strategy for the primary prevention of EC in women at elevated risk.

Awareness of the strong association between obesity and EC remains suboptimal, both among patients and within gynecologic oncology training and clinical practice. Despite the epidemiologic evidence, a 2008 survey reported that 58% of healthy women were unaware that obesity increases the risk of EC [15]. More recent data indicate that only one third of survivors recall receiving counseling on weight loss during gynecologic oncology visits [16]. Patients who are informed about obesity-related cancer risks are more likely to recognize weight loss as a key component of overall health and to demonstrate greater adherence to therapeutic recommendations compared with those who lack such awareness. In response to this gap, the Society of Gynecologic Oncology (SGO) has developed an Obesity Toolkit to support gynecologic oncology providers in addressing obesity and weight management with their patients [17]. Additionally, multidisciplinary quality improvement initiatives for both patients and gynecologic oncologists have been shown to effectively increase discussions about obesity and referrals to weight-loss clinics in patients with low-risk EC [16].

Beyond clinical awareness and management, obesity also has significant economic implications in the context of EC care, increasing healthcare costs through several mechanisms. Obese and morbidly obese patients have longer hospital stays, higher rates of perioperative complications, and require more intensive care, including mechanical ventilation [18]. In the United States, morbidly obese patients undergoing hysterectomy for EC incur median hospital charges which are approximately $5000 higher per patient, after adjustment for confounders [18]. As rates of obesity continue to rise in younger age groups, the onset of EC occurs earlier, resulting in a higher incidence among premenopausal women who may require fertility-sparing treatments, further increasing costs and complexity. Previous data have suggested that younger women (<45 years) diagnosed with EC are significantly more obese than older patients diagnosed with the same cancer, and also experience higher morbidity and overall mortality [19].

## 4. Innovations in Metabolic Bariatric Surgery Reframing Endometrial Cancer Treatment

Metabolic bariatric surgery has been demonstrated to be the most effective treatment for individuals with severe obesity who are not responsive to non-surgical approaches, resulting in a sustained loss of approximately 25 to 30% of total body weight [20]. Beyond simple weight reduction, bariatric surgery directly induces a series of physiological and endocrine modifications that mediate its therapeutic effects in obese patients, leading to decreased ghrelin production and increased GLP-1 and peptide YY (PYY) secretion as a consequence of the accelerated delivery of nutrients to the distal ileum [21]. These surgery-driven endocrine shifts enhance satiety, retard gastric emptying, and stimulate glucose-dependent insulin secretion, thereby yielding sustainable improvements in glycemic control and, in many cases, remission of type 2 diabetes [22].

**Figure 2:**
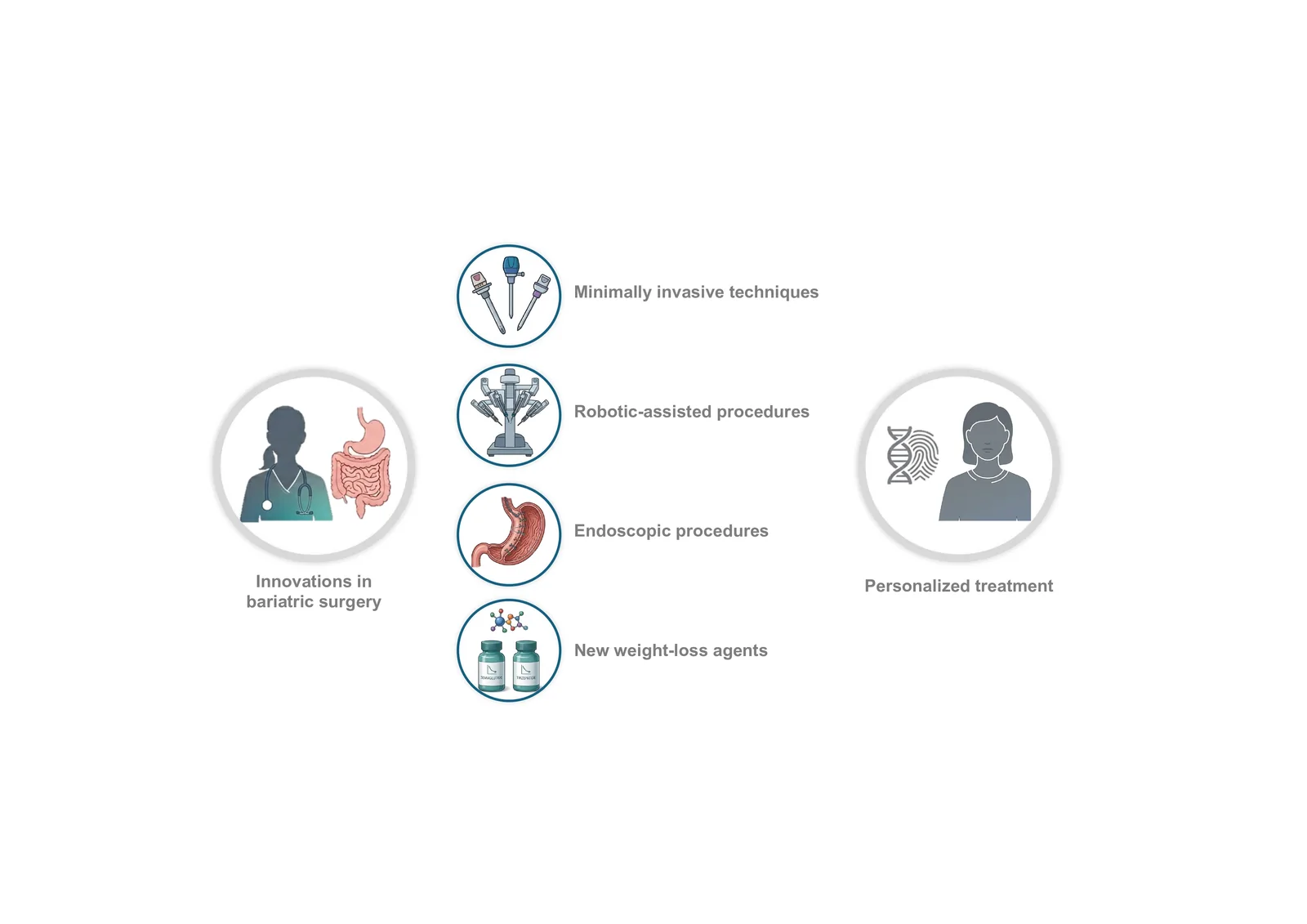
Key innovations in metabolic bariatric interventions.

There are many types of bariatric intervention, such as gastric banding, Roux-en-Y gastric bypass (RYGB), and sleeve gastrectomy, each with distinct advantages and disadvantages. Although laparoscopic surgery remains the gold standard, technological innovations are progressively moving toward less invasive, organ-preserving, and easily reproducible interventions [23] (Figure 2). Endoscopic sleeve gastroplasty (ESG) is an example of this shift. As a fully endoscopic procedure that reduces gastric volume via transmural suturing, ESG delivers clinically meaningful weight loss with a favorable safety profile, representing a compelling option for patients with class I–II obesity or those ineligible for surgical intervention [24]. Notably, the feasibility of performing ESG under deep sedation rather than general anesthesia represents a significant advancement, enabling shorter operative times, higher rates of ambulatory management, lower postoperative resource utilization, and comparable weight-loss outcomes in appropriately selected patients [25]. Concurrently, robotic-assisted bariatric surgery is gaining traction for its potential to enhance precision, ergonomics, and reproducibility in revisional procedures and anatomically complex cases [26]. In RYGB, robotic assistance may confer technical advantages in anastomotic construction; however, evidence of superior clinical outcomes remains inconsistent, and operative costs are substantially higher [27]. In sleeve gastrectomy, procedural standardization limits the added value of robotic approaches, which have not demonstrated significant perioperative benefit over conventional laparoscopy. Accordingly, laparoscopy retains its status as the reference standard, while robotic surgery is reserved for high-complexity cases within experienced centers [26].

Beyond metabolic and cardiovascular benefits, bariatric surgery is increasingly recognized as a potential cancer prevention strategy for patients with obesity. Observational evidence and meta-analyses consistently demonstrate a significant reduction in overall cancer incidence and cancer-related mortality following bariatric procedures compared with non-surgical management [28], particularly for hormonally driven cancers such as breast cancer and EC, as well as selected gastrointestinal and hepatobiliary cancers [29]. Prospective data indicate that weight loss is associated with attenuated systemic inflammation and with the recruitment of protective CD8+ cytotoxic T cells to the endometrium, supporting antitumor surveillance and promoting the natural clearance of precursor and precancerous lesions [30]. Through the mitigation of hyperinsulinemia and hyperleptinemia, bariatric surgery may exert a protective effect on the endometrium, potentially reducing susceptibility to malignant transformation and disease progression. Retrospective evidence suggests an association between bariatric surgery and reduced risk of EC, with some studies reporting reductions of up to 70% [31], consistent with the results of the SPLENDID study, an observational matched-cohort analysis, that showed a significant reduction in risk of EC after surgery compared with the non-surgical control group [32]. A prospective study in 72 women with severe obesity, for whom endometrial biopsies were examined before and after bariatric surgery, showed a significant reduction in the markers of endometrial proliferation and oncogenic signaling after surgery, through the down-regulation of PI3K-AKT-mTOR signaling [33].

Early evidence suggests that the combination of bariatric surgery with minimally invasive hysterectomy may be associated with improvements in underlying metabolic disease, postoperative weight loss, long-term related mortality, and quality of life in patients with EC and obesity [34]. Evidence of reduced cancer incidence after bariatric surgery cannot be directly extrapolated to improved oncologic outcomes after an established diagnosis of endometrial cancer, as primary prevention and the treatment of active disease represent distinct clinical settings.

As summarized in Table 1, the integration of metabolic bariatric surgery into the management of carefully selected patients with early-stage disease has been explored in a limited number of prospective and observational experiences. These reports indicate the potential for histologic regression in patients undergoing fertility-sparing treatment, the technical feasibility of simultaneous bariatric surgery and minimally invasive hysterectomy, and the possibility of deferring hysterectomy for several months, particularly when interim hormone therapy is administered [35,36,37,38,39,40]. However, currently available evidence remains narrow and is largely derived from case reports and small case series involving highly selected patients treated in expert, high-volume centers. As reflected in Table 1, most patients had early-stage, low-grade endometrioid tumors, relatively young age, severe obesity (BMI ranging from 40.7 to 95.0 kg/m^2^), and a high burden of metabolic comorbidities and were managed within multidisciplinary settings. This introduces significant selection bias and limits the external validity of the reported outcomes.

Different management strategies were described, including sequential, combined, and conservative approaches with hormonal therapy and hysteroscopic surveillance. A combined synchronous surgical approach may be appropriate for patients with early-stage disease in whom immediate definitive surgery is indicated and surgical risk is deemed acceptable, allowing a single anesthetic event and potentially faster metabolic benefit [38]. In contrast, a sequential strategy, with bariatric surgery preceding oncologic treatment, may be considered in selected patients undergoing fertility-sparing management or in those with high perioperative risk, where preoperative weight loss and metabolic optimization could improve subsequent surgical safety [37].

Notably, sleeve gastrectomy, performed through either laparoscopic or robotic approaches, represents the most frequently performed bariatric procedure in the available literature, while Roux-en-Y gastric bypass is less commonly adopted [36]. Endoscopic techniques remain less explored; however, an ongoing prospective observational study (NCT07397624) is currently investigating the safety and feasibility of endoscopic suture gastroplasty in obese patients with EC. Although the limited methodological quality and heterogeneity of the existing evidence preclude definitive conclusions, the authors consistently report achievement of clinically meaningful weight loss alongside improvement in metabolic parameters following bariatric surgery, when performed prior to oncologic treatment. Oncologic outcomes appear similarly encouraging, with reported disease-free survival and an absence of recurrences during follow-up periods extending up to 41 months, though these findings warrant cautious interpretation in the absence of controlled comparative data; furthermore, delaying hysterectomy, even in low-risk disease, raises important oncologic considerations that remain insufficiently addressed. Furthermore, bariatric surgery in young women of reproductive age carries specific considerations, including nutritional deficiencies, particularly of iron, folate, and vitamin B12, that may adversely affect reproductive outcomes and require careful preconception planning and long-term nutritional monitoring [41]. Overall, whether the observed metabolic and perioperative benefits translate directly into improved cancer-specific outcomes remains to be established in prospective, large-cohort studies.

Combined bariatric–oncologic surgery should be regarded as a highly selected strategy for expert centers with both gynecologic oncology and bariatric expertise, rather than a broadly generalizable standard of care. From a health economics perspective, modeling analyses have suggested that combined bariatric–oncologic surgery may reduce projected lifetime healthcare costs relative to hysterectomy alone, potentially strengthening the rationale for its adoption [42,43]. Nevertheless, the practical implementation remains constrained by the requirement for highly specialized multidisciplinary expertise and substantial logistical infrastructure, factors that are likely to limit its widespread applicability in routine clinical settings. For this reason, an ongoing clinical trial (NCT04839614) is investigating the feasibility and potential benefits of concurrent hysterectomy and bariatric surgery in patients with endometrial intraepithelial neoplasia (EIN) or EC, whereas a separate trial (NCT04008563) evaluates bariatric surgery combined with progestin therapy in the fertility-sparing setting, both aiming to optimize oncologic outcomes and quality of life. Consequently, while recognizing the potential preventive role of metabolic intervention in selected populations, early referrals for bariatric surgical assessment should be considered within a multidisciplinary framework for young patients with severe metabolic disease and endometrial pathology, pending the availability of more robust evidence.

## 5. Novel Weight-Loss Medications in Endometrial Cancer

Metformin has long been used in weight management and is considered a treatment option for EC due to its antitumorigenic activity through indirect effects on the metabolic milieu (decreased insulin and glucose levels) and direct effects on the tumor through inhibition of mitochondrial complex 1 and subsequent AMPK activation/mTOR inhibition, as well as decreased fatty acid and lipid synthesis [44]. Despite some mixed findings, the combination of metformin with progestin has demonstrated promise in reversing endometrial hyperplasia (EH) and enhancing outcomes for EC patients [45]; however, its addition to standard-of-care paclitaxel and carboplatin in patients with advanced and recurrent EC did not show significant improvements in progression-free survival and overall survival [44].

Newer agents such as the agonists of the glucagon-like peptide-1 receptor (GLP-1RAs) have emerged as effective treatments for T2D and obesity. Although the exact mechanism is unknown, the anorexigenic effect is thought to be mediated through delayed gastric emptying, reduced hunger, and enhanced satiety via central hypothalamic pathways [46,47]. Given the widespread expression of GLP-1R and the established link between obesity and cancer, evidence has begun to characterize the potential for GLP-1RAs to modulate cancer development and progression. A large retrospective cohort study reported a reduced overall cancer risk among GLP-1RA users compared with non-users, with significant risk reductions observed for endometrial, meningioma and ovarian cancers and a nonsignificant increase in kidney cancer risk [48]. These results are consistent with a nationwide cohort study of 1.6 million patients with type 2 diabetes, in which the use of GLP-1RAs was associated with a 26% lower endometrial cancer risk compared with insulin, with no significant difference compared with metformin [49]. Conversely, a meta-analysis of fifty RCTs found no statistically significant association between GLP-1RA treatment and overall cancer incidence [50].

Given that more than 799 million individuals worldwide are currently eligible for GLP-1RA therapies, even modest changes in cancer risk could have substantial public health implications [51], but current evidence is insufficient to conclude that GLP-1RAs exert a direct antitumor effect. Risk, or incidence, refers to the development or diagnosis of cancer, which is influenced by several factors, including genetic predisposition, environmental exposure, and lifestyle. A critical and largely unresolved confounding factor is weight loss itself: GLP-1RA therapies induce meaningful reductions in body weight, and weight loss, whether achieved through lifestyle modification, pharmacotherapy, or bariatric surgery, is independently associated with reduced risk for several obesity-related cancers, including breast and endometrial cancers. Furthermore, GLP-1RAs attenuate basal hyperinsulinemia, which is itself an established risk factor for endometrial, pancreatic, and breast cancers. Consequently, the cancer risk reductions observed in epidemiological studies may be substantially attributable to these downstream metabolic effects rather than to any direct receptor-mediated antineoplastic activity.

The effects of these drugs specifically on the endometrium need further investigation, and currently, only preclinical data support an antitumor effect of GLP-1RAs in endometrial cancer. In vivo, exenatide has been reported to attenuate EC cell growth, and semaglutide to enhance the anticancer effect of levonorgestrel, when used in combination [52,53]. Mechanistically, GLP-1RAs increase GLP-1R and progesterone receptor (PR) expression, enhancing signaling via PGRMC1–Src/ERK-mediated phosphorylation, and drive transcription of antiproliferative/apoptotic genes (e.g., p21, p27, and Bak), thereby reducing tumor cell viability [54]. These findings derive from preclinical models; GLP-1R expression has not been validated as a predictive tissue biomarker or as a determinant of treatment response in endometrial cancer and should not be interpreted as such.

The integration of GLP-1RAs into fertility-sparing management strategies for early-stage endometrial cancer and AEH/EIN (atypical endometrial hyperplasia/endometrial intraepithelial neoplasia) is supported by a biological rationale; however, translating these findings to clinical practice may prove premature. Current standard-of-care recommends oral and/or intrauterine progestin-based therapies for patients with atypical endometrial hyperplasia/endometrial intraepithelial neoplasia or low-grade, early-stage endometrial carcinoma who desire fertility preservation, as well as for medically inoperable patients [12]. Given that response to hormonal therapy is often protracted, typically requiring 6–12 months or longer to achieve complete regression, this therapeutic interval may provide a critical window for metabolic optimization and weight reduction. GLP-1RAs could support fertility preservation by reducing adiposity and improving metabolic and inflammatory profiles, thereby increasing the likelihood of future pregnancy. Nonetheless, safety concerns persist, including evidence suggesting a potential link between GLP-1RAs and kidney malignancies, and clinical experience in gynecologic oncology remains limited [55,56].

A retrospective cohort analysis found that adding GLP-1RAs to progestin therapy (LNG-IUD and systemic progestins) was associated with reduced progression to endometrial cancer in women with EH or benign uterine pathology, compared with progestin alone, and GLP-1RAs plus LNG-IUD was also associated with a greater risk reduction than metformin plus LNG-IUD [57]. However, several limitations intrinsic to the retrospective observational nature of the study limit causal inference; therefore, these findings remain hypothesis-generating. Prospective studies are needed to define oncologic safety, reproductive benefits, optimal integration relative to progestin therapy, and the patient subgroups most likely to derive clinically meaningful benefits.

To date, no study has specifically evaluated GLP-1RAs for preoperative weight loss before endometrial cancer surgery. Ongoing and recently registered studies evaluating metabolic interventions across the fertility-sparing, preoperative, surgical, and survivorship settings are summarized in Table 2.

Future trials should methodologically control for weight-loss-related confounding and be designed to disentangle direct drug-related effects from indirect benefits mediated by weight loss, improved insulin sensitivity, reduced inflammation, immunomodulation and changes in estrogen bioavailability, using standardized oncologic and metabolic endpoints in survivorship settings [54].

## 6. Future Directions Toward a Personalized Metabolic and Oncologic Approach in Endometrial Cancer Care

**Figure 3:**
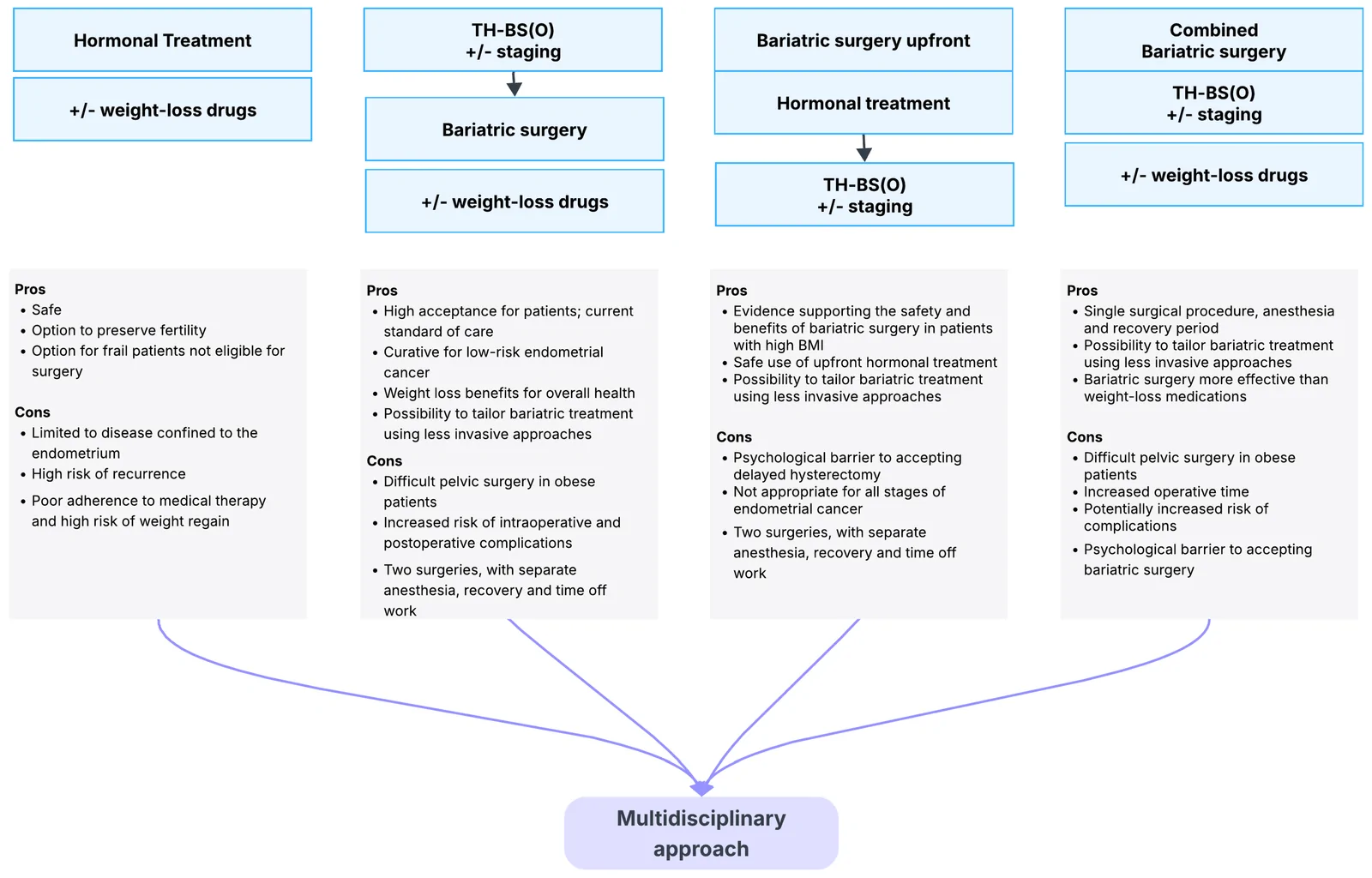
Integrated multidisciplinary approach to endometrial cancer and obesity: benefits and pitfalls (TH-BS(O), total hysterectomy with bilateral salpingo-oophorectomy).

The rising incidence and mortality of EC are increasingly driven by the global obesity epidemic, and the healthcare burden will continue to escalate unless obesity is addressed in parallel [58]. Acknowledging that obesity is a chronic disease underscores the need to integrate sustainable weight management strategies into comprehensive oncologic care for patients with EC and neoplastic precursors (Figure 3).

### 6.1. Patients Eligible for Fertility-Sparing Management

In the population eligible for fertility-preserving strategies, the use of GLP-1RAs in synergy with progestins is a promising approach; ongoing trials will clarify clinical utility, and next-generation incretins (dual and triple incretin-based co-agonists) may provide further evidence. Based on individual patient characteristics and treatment availability, weight reduction in young patients may also be effectively achieved through bariatric surgery [59], which has also been associated with improved pregnancy outcomes and a reduced risk of intraoperative and postoperative complications during the subsequent oncologic surgery, which remains guideline-recommended following the completion of fertility-sparing management [60]. Conventional eligibility for fertility-sparing management is restricted to grade 1 endometrioid carcinoma of FIGO stage IA without myometrial invasion, whereas conservative management of grade 2 disease should be regarded as an individualized option supported by limited evidence. Given the recognized diagnostic variability between AEH/EIN and endometrioid carcinoma, the initial diagnosis should be reviewed by an experienced gynecologic pathologist before conservative treatment is undertaken.

### 6.2. Patients Not Eligible for Fertility Preservation

A different paradigm applies to patients who are not candidates for fertility preservation, in whom increasing attention is being directed toward combining hysterectomy with metabolic bariatric interventions [34]. However, combined oncologic and bariatric procedures carry inherent surgical risks, including anastomotic complications, nutritional deficiencies, dumping syndrome and procedure-specific morbidity [55]. Advances in robotic and minimally invasive endoscopic techniques enable personalized treatment strategies tailored to patient-specific risk profiles. As the efficacy and complication rates of bariatric procedures vary by technique, combinations of lower-risk interventions, such as endoscopic bariatric procedures, with early pharmacologic therapy represent an attractive and underexplored strategy, particularly for younger patients and frail populations [21].

### 6.3. Cancer Survivors

Obesity may exert a greater influence on long-term health outcomes and longevity of cancer survivors than in the general population [8]. It can indirectly compromise treatment efficacy by locally and systemically reshaping the immune microenvironment, thereby promoting resistance to chemotherapy, radiotherapy, and targeted therapies [61]. Pharmacologic weight-loss therapies may be beneficial for patients who are not suitable candidates for bariatric surgery or who require adjuvant treatments, such as radiotherapy and/or chemotherapy, following hysterectomy. In these settings, they offer a non-surgical approach that can be generally implemented without delaying the oncologic treatment pathway, thereby supporting both metabolic health and timely cancer care. Notably, treatment discontinuation is associated with significant weight regain, and clinicians should therefore frame GLP-1RA therapy as a long-term commitment requiring ongoing monitoring and active management [62].

**Figure 4:**
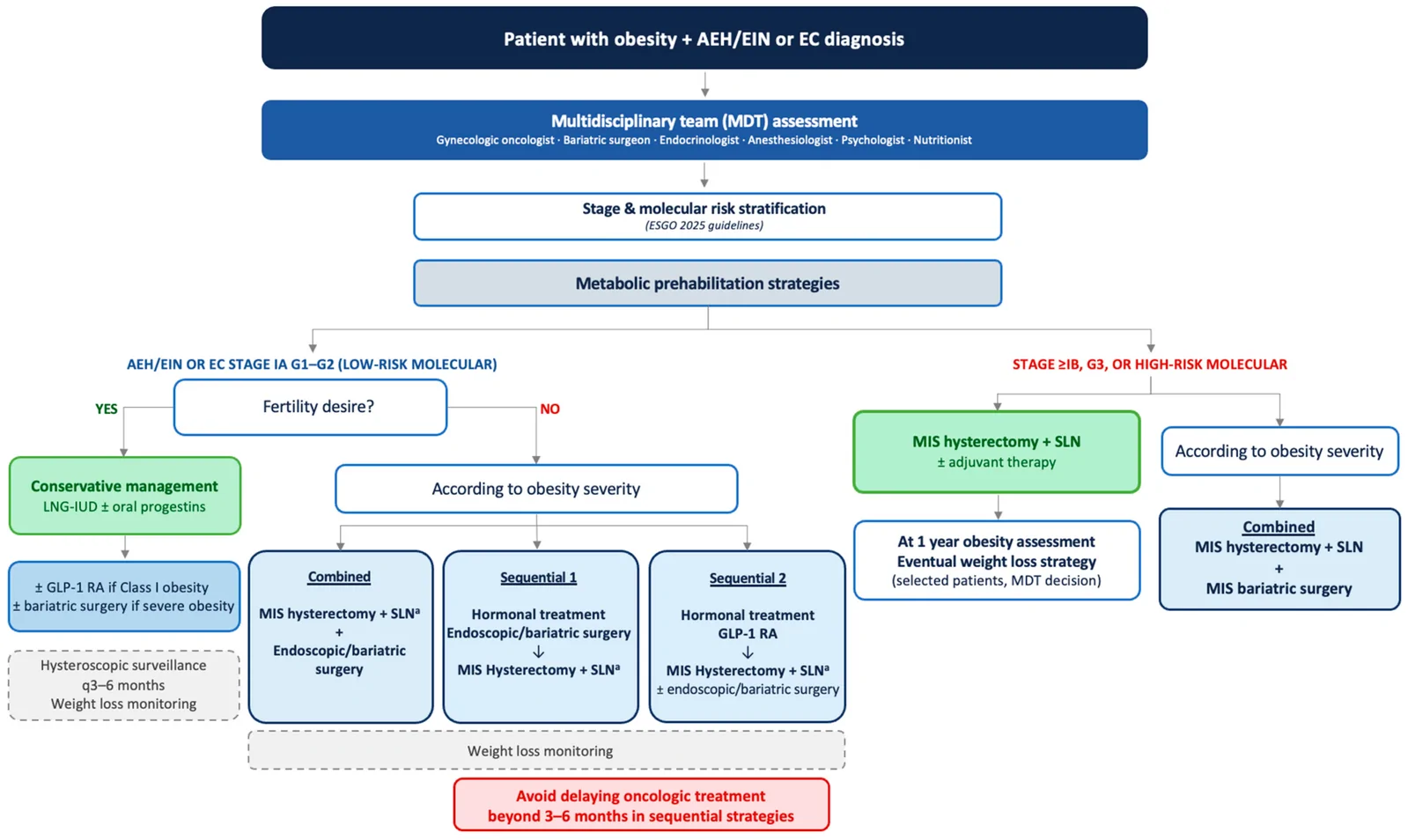
Proposed hypothesis-generating research framework for metabolic strategies in endometrial cancer management. Box colors indicate the level of supporting evidence: green boxes denote standard-of-care options, whereas light-blue boxes denote investigational approaches supported only by observational data, case reports, expert opinion, or ongoing experimental studies. (AEH/EIN, atypical endometrial hyperplasia/endometrial intraepithelial neoplasia; BMI, Body Mass Index; EC, Endometrial Cancer; GLP-1RA, GLP-1 Receptor Agonist; MIS, Minimally Invasive Surgery; MDT, Multidisciplinary Tumor Board; LVSI, Lymphovascular Space Invasion; SLN, Sentinel Lymph Node; ^a^ the SLN procedure is performed only when appropriate.). In this framework, “low-risk” denotes an integrated clinicopathological and molecular assessment (histotype, grade, FIGO stage, LVSI and molecular class); sentinel lymph node assessment applies only to confirmed carcinoma.

The coordination of these complex care pathways requires a multidisciplinary approach and well-informed, actively engaged patients. The current literature remains insufficient to establish universally applicable selection criteria. As a pragmatic step, we propose a preliminary framework (Figure 4) to guide individualized therapeutic decision-making, acknowledging that clinical judgment must continue to account for several unresolved challenges: the limited availability of expert centers with the multidisciplinary expertise required to safely manage complex interventions in patients who are frequently frail and oncologically compromised, and the cautious integration of emerging agents such as GLP-1RAs, whose long-term safety profile in this specific population and potential interactions with oncologic treatments have yet to be fully characterized. This framework should be regarded as a hypothesis-generating research proposal, distinguishing standard-of-care options from approaches supported only by observational data, case reports, expert opinion, or ongoing experimental studies.

The development of risk-based stratification tools, also comprising EC molecular profiling, may further enable personalized treatment selection, allowing less aggressive interventions to be more tailored to patient characteristics [54]. Molecular classification according to The Cancer Genome Atlas (TCGA) distinguishes four prognostic groups—POLE-ultramutated, mismatch repair-deficient/microsatellite-unstable, no specific molecular profile, and p53-abnormal—that are increasingly incorporated into risk stratification. These molecular findings should be interpreted together with histological subtype, grade, FIGO stage, and lymphovascular space invasion (LVSI). At present, there is insufficient evidence that the response to metabolic bariatric surgery or to GLP-1RAs differs across these molecular subtypes.

## 7. Practical, Ethical and Access-Related Considerations

Several important practical, ethical, and structural barriers currently limit the widespread implementation of metabolic interventions in the management of endometrial cancer. From a clinical standpoint, integrating bariatric surgery into oncologic care pathways introduces inherent procedural risks and long-term nutritional deficiencies that require sustained multidisciplinary monitoring [63]. The ethical complexity of deferring definitive cancer treatment after bariatric intervention raises concerns about disease progression that remain insufficiently addressed in the available literature, given the absence of controlled prospective data. Weight regain following bariatric surgery, although less pronounced than with pharmacotherapy, is an additional challenge, particularly in the long-term maintenance of metabolic benefits.

Regarding pharmacotherapy, weight regain following pharmacotherapy discontinuation represents a clinically important limitation that tempers the long-term effectiveness of GLP-1RAs in this population [64]. Furthermore, limited long-term safety data preclude definitive conclusions about their integration into standard oncologic protocols. Access and equity represent equally critical concerns. GLP-1RAs remain costly and are not universally reimbursed across healthcare systems, creating significant disparities in availability based on socioeconomic status and geographic context [65]. Addressing disparities in access and conducting comprehensive health economic analyses is critical to ensure equitable benefit and inform healthcare policy and resource allocation. Similarly, the implementation of combined bariatric–oncologic surgery requires highly specialized multidisciplinary expertise and institutional infrastructure that is concentrated in a limited number of high-volume centers, restricting equitable access for the broader patient population. These limitations are expected to attenuate as prospective evidence accumulates, reimbursement policies evolve, and specialized centers expand their reach; however, they must be explicitly acknowledged when interpreting and translating current findings into clinical practice.

## 8. Conclusions

Multidisciplinary weight management strategies can significantly reduce both the incidence of and mortality from obesity-related cancers, including endometrial cancer. Accordingly, obesity should be regarded not only as a major risk factor but also as a modifiable therapeutic target that can be integrated into preventive and therapeutic interventions across different stages of the cancer care pathway. When integrated within preoperative windows of opportunity, weight-loss prehabilitation may improve surgical feasibility and oncologic outcomes while preserving the timeliness of cancer treatment. Prospective trials and formal guidelines are needed to define appropriate patient selection, timing and modality of intervention, calling for careful multidisciplinary coordination, ethical oversight, and equitable access to care.

## Figures and Tables

**Table 1:** Studies integrating bariatric surgery into the management of obese patients with early-stage endometrial cancer.

Authors + Year	Country	Study	N. of Patients	Mean Age ± SD (Years)	Mean Body Weight (kg)	Mean BMI ± SD (kg/m^2^) at the Time of Cancer Surgery	Comorbidities	Preoperative Hemoglobin A1c (%)	Endometrial Pathology Histotype and Grade	FIGO Stage (2009)	Hormonal Treatment	Weight-Loss Medications	Type of Bariatric Surgery	Type of Gynecologic Oncologic Surgery	Timing of Surgeries	Interval Between Surgeries	Reported Disease-Free Duration	Follow-Up After Bariatric Intervention (Months)
Montemorano et al., 2019 [35]	United States	Case report	1	30	ND	95.0	anxiety, depression	ND	Endometrioid G1	IA	Megestrol 80 mg bid	NA	LPS Sleeve gastrectomy	RA THBSO	Sequential: bariatric before oncologic	9 months	41 months	After 8 months: BMI reduction of 28.2%
Shafa et al., 2019 [36]	United States	Case report	1	54.0	133.9	50.0	type 2 diabetes mellitus, neuropathy, retinopathy	7.3%	Endometrioid G1	IA	NA	NA	LPS Roux-en-Y gastric bypass	RA THBSO	Combined	/	12 months	After 6 months: hemoglobin A1c 6.9% After 12 months: 30.8% weight loss
Lin et al., 2023 [37]	Singapore	Case series	5	32.0	109.5	40.7	asthma, hypertension, diabetes mellitus, hyperlipidemia	ND	Endometrioid G1	IA	Megestrol, triptorelin, leuprolide, levonorgestrel-releasing IUD	NA	LPS Sleeve gastrectomy	FST: HDC on three monthly intervals for surveillance	Sequential	/	12 months Complete EC regression in 80% of patients. Regressed to simple hyperplasia in 10%.	After 3–19 months: mean total weight loss of 27.24 kg, mean percentage total weight loss of 25.28%
Mezzapesa et al., 2024 [38]	Italy	Case series	5 (4 completed combined surgery)	54.8 ± 9.09	143.9	49.7	type 2 diabetes mellitus (15.4%), hypertension (61.5%), chronic respiratory failure, obstructive sleep apnea syndrome (23%) ^a^	ND	Endometrioid G1 (20%), G2 (20%), G3 (60%)	IA (40%) IB (40%) II (20%)	NA	NA	RA Sleeve gastrectomy	RA THBSO + SLN	Combined	/	24 months	After 6 months ^b^: mean BMI 41.14 (± 11.22), ΔBMI − 8.53 ± 7.16 (p 0.016) After 24 months: 43.8 kg mean weight loss
Ege et al., 2024 [39]	Turkey	Case report	1	40.0	ND	62.4	type II diabetes mellitus, retinitis pigmentosa	ND	Endometrioid	IA	NA	NA	RA Sleeve gastrectomy	RA THBSO	Combined	/	ND	ND
Arab et al., 2024 [40]	Iran	Case report	2	43.5	ND	51.8	n.1: hypothyroidism, polycystic ovarian disease, eight years of infertility n.2: diabetes, hypertension, ischemic heart disease, liver cirrhosis	ND	Endometrioid G1	IA	Megestrol 80 mg bid	NA	LPS Sleeve gastrectomy	LPS THBSO + PLND	Sequential: bariatric before oncologic	n.1: 9 months n.2: 4 months	35 months	n.1: 35 months after sleeve gastrectomy ΔBMI—18.1 n.2: 24 months after sleeve gastrectomy ΔBMI—18.3

EC, endometrial cancer; FIGO, International Federation of Gynaecology and Obstetrics; FST, fertility sparing treatment; HDC, hysteroscopy, dilation, and endometrial curettage; IUD, intrauterine device; LPS, laparoscopic; NA, not administered; ND, not determined; PLND, pelvic lymphadenectomy; RA, robotic assisted; SLN, sentinel lymph node; THBSO, total hysterectomy and bilateral salpingo-oophorectomy. ^a^ Comorbidities referred to the entire cohort; ^b^ 6-month BMI and ΔBMI values derive from the intention-to-treat analysis, which included one patient whose sleeve gastrectomy was delayed.

**Table 2:** Ongoing and registered clinical studies of metabolic interventions in endometrial cancer and precursor lesions.

Trial (ID/Acronym)	Clinical Setting	Intervention	Design/Phase	Population	Primary Outcome	Status
NCT05829460	Fertility-sparing	Semaglutide + LNG-IUD vs. placebo + LNG-IUD	Phase II RCT, double-blind, placebo-controlled	Premenopausal, obesity, AEH/EIN	Atypia-free biopsy with uterine preservation at 2 years	Withdrawn (low accrual)
NCT06073184 (WE-FiERCE)	Fertility-sparing	Tirzepatide + progestin	Phase II, single-arm, open-label	Obesity, AEH/EIN or grade 1 endometrioid EC	Complete pathologic response	Not yet recruiting
NCT04008563 (B-FiERCE)	Fertility-sparing	Bariatric surgery + progestin IUD vs. progestin IUD alone	Randomized, open-label	Obesity, AEH/EIN or grade 1 EC desiring fertility preservation	Recruitment rate	Recruiting
NCT07065552	Preoperative window	Short preoperative course of tirzepatide before hysterectomy	Window-of-opportunity, single-arm	Obesity, endometrial cancer	Reduction in tumor cell proliferation	Recruiting
NCT07078838	Preoperative window	Tirzepatide vs. no tirzepatide, ~4 weeks before hysterectomy	Randomized, open-label	Obesity (BMI > 30), EIN or grade 1 EC	Reduction in tumor cell proliferation	Not yet recruiting
NCT04839614	Concurrent hysterectomy + bariatric surgery	Concurrent MIS hysterectomy + weight-loss (bariatric) surgery	Interventional, feasibility	Obesity, EC or EIN	Feasibility of expedited concurrent surgery	Recruiting
NCT07397624	Bariatric endoscopy	Endoscopic suture gastroplasty (ESG)	Prospective, multicenter, observational	Obesity, endometrial cancer	Safety/feasibility, weight loss, metabolic profile	Recruiting
NCT06751589	During adjuvant chemotherapy	Tirzepatide or semaglutide	Phase I	Stage I–III EC with overweight/obesity/diabetes receiving adjuvant chemotherapy after surgery	Feasibility of weight management	Recruiting
NCT06877572	Survivorship	Multidisciplinary weight management (incl. optional weight-loss pharmacotherapy)	Prospective observational	Survivors of low-risk, early-stage EC, BMI ≥ 30	Implementation/clinical outcomes	Recruiting

AEH/EIN, atypical endometrial hyperplasia/endometrial intraepithelial neoplasia; BMI, body mass index; EC, endometrial cancer; ESG, endoscopic suture gastroplasty; LNG-IUD, levonorgestrel-releasing intrauterine device; MIS, minimally invasive surgery; RCT, randomized controlled trial.

## Data Availability

All data generated or analyzed in this review are included in this article and/or its figures. Further inquiries may be directed to the corresponding authors.
